# Targeting Fatty Acid Synthase Modulates Metabolic Pathways and Inhibits Cholangiocarcinoma Cell Progression

**DOI:** 10.3389/fphar.2021.696961

**Published:** 2021-08-04

**Authors:** Jittima Tomacha, Hasaya Dokduang, Sureerat Padthaisong, Nisana Namwat, Poramate Klanrit, Jutarop Phetcharaburanin, Arporn Wangwiwatsin, Tueanjit Khampitak, Supinda Koonmee, Attapol Titapun, Apiwat Jarearnrat, Narong Khuntikeo, Watcharin Loilome

**Affiliations:** ^1^ Department of Biochemistry, Faculty of Medicine, Khon Kaen University, Khon Kaen, Thailand; ^2^ Cholangiocarcinoma Research Institute, Khon Kaen University, Khon Kaen, Thailand; ^3^ Department of Pathology, Faculty of Medicine, Khon Kaen University, Khon Kaen, Thailand; ^4^ Department of Surgery, Faculty of Medicine, Khon Kaen University, Khon Kaen, Thailand

**Keywords:** lipid metabolism1, acetyl-CoA carboxylase2, fatty acid synthase3, HMG-CoA reductase4, metabolomics5, cholangiocarcinoma6

## Abstract

An aberrant regulation of lipid metabolism is involved in the pathogenesis and progression of cancer. Up-regulation of lipid biosynthesis enzymes, including acetyl-CoA carboxylase (ACC), fatty acid synthase (FASN) and HMG-CoA reductase (HMGCR), has been reported in many cancers. Therefore, elucidating lipid metabolism changes in cancer is essential for the development of novel therapeutic targets for various human cancers. The current study aimed to identify the abnormal expression of lipid-metabolizing enzymes in cholangiocarcinoma (CCA) and to evaluate whether they can be used as the targets for CCA treatment. Our study demonstrated that a high expression of FASN was significantly correlated with the advanced stage in CCA patients. In addition, survival analysis showed that high expression of FASN and HMGCR was correlated with shorter survival of CCA patients. Furthermore, FASN knockdown inhibited the growth, migration and invasion in CCA cell lines, KKU055 and KKU213, as well as induced cell cycle arrest and apoptosis in the CCA cell lines. In addition, metabolomics study further revealed that purine metabolism was the most relevant pathway involved in FASN knockdown. Adenosine diphosphate (ADP), glutamine and guanine levels significantly increased in KKU213 cells while guanine and xanthine levels remarkably increased in KKU055 cells showing a marked difference between the control and FASN knockdown groups. These findings provide new insights into the mechanisms associated with FASN knockdown in CCA cell lines and suggest that targeting FASN may serve as a novel CCA therapeutic strategy.

## Introduction

Lipid metabolism is the biosynthesis and degradation processes of lipids in cells, relating to the storage or breakdown of fats for energy and the synthesis of functional and structural lipids. An aberrant regulation of lipid metabolism is involved in the pathogenesis and progression of cancer. Various evidence reported that an upregulation of lipid biosynthesis enzymes, including acetyl-CoA carboxylase (ACC), fatty acid synthase (FASN) and HMG-CoA reductase (HMGCR), can be found in many cancer types. For example, the upregulation of ACC contributes to the cell proliferation and migration of liver cancer. The overexpression of ACC in liver cancer tissues was correlated with a poorer prognosis and shorter survival for liver cancer patients. Moreover, down-regulation of ACC protein expression using siRNA leads to decreased liver cancer cell growth and migration (Ye et al., 2019). In addition, the up-regulation of FASN enhances colorectal cancer cell proliferation and metastasis. The high expression of FASN in colorectal cancer tissues was correlated with lymph node metastasis, the Tumor, Node, Metastases (TNM) stage and poor prognosis in colorectal cancer patients. Furthermore, FASN knockdown resulted in reduced colorectal cancer cell proliferation and migration while FASN overexpression had the opposite effects on colorectal cancer cells (Lu et al., 2019). The up-regulation of HMGCR can promote gastric cancer cell growth and migration. HMGCR overexpression is found in gastric cancer tissues and cell lines. In contrast, HMGCR knockdown can inhibited gastric cancer cell growth and migration both *in vitro* and *in vivo* (Chushi et al., 2016). These data demonstrate that ACC, FASN and HMGCR are promising potential targets for cancer treatment. Therefore, elucidating lipid metabolism changes in cancer is required to develop therapeutic targets for various human cancers.

Metabolomics is a systems biology tool for studying biochemical composition and investigating metabolic pathway alteration within an organism, cell or tissue. Metabolomics has been used widely in cancer research to explore potential biomarkers for early detection and diagnosis; for example in colorectal cancer (Zhang et al., 2014) and ovarian cancer (Gaul et al., 2015). It is also useful for providing better understanding of the molecular mechanisms (Griffin & Shockcor, 2004). In this study, liquid chromatography-mass spectrometry (LC-MS) was used to conduct metabolomics profiling. Because of its high sensitivity and selectivity, LC-MS is superior in secondary metabolite analysis at the detection level of picomole to femtomole (Emwas, 2015). The study of metabolomics provides new insights into the metabolic processes within cells and can be used to determine biomarkers for novel cancer therapeutic strategies.

Cholangiocarcinoma (CCA) is a bile duct cancer that is caused by malignant transformation of the cholangiocytes. CCA tumors are classified according to the position of tumor along the biliary tract and comprises intrahepatic CCA (iCCA), perihilar CCA (pCCA) and distal CCA (dCCA) (Alsaleh et al., 2019). Although CCA is a rare disease in many countries, the highest incidence has been reported in the northeast of Thailand (Khuntikeo et al., 2015). A major risk factor of CCA development in this area is related to chronic inflammation induced by liver fluke *Opisthorchis viverrini* infection, which leads to the alteration of genes, proteins and molecules such as increased proinflammatory cytokines levels and overproduction of reactive oxygen and nitrogen species (Yongvanit et al., 2012). Moreover, CCA is asymptomatic in its early stage and most patients are diagnosed when the disease becomes advanced resulting in short survival post-treatment and a poor prognosis (Banales et al., 2016). However, there is very limited information on lipid metabolism in CCA. Therefore, an in-depth study on lipid metabolism in CCA is required in order to improve patient survival and prognoses. In the present study, we aimed to identify the abnormal expression of lipid-metabolizing enzymes in CCA and to evaluate their potential as the targets for CCA treatment.

## Materials and Methods

### Human Cholangiocarcinoma Tissues and Cell Lines

One hundred fifty-five formalin-fixed, paraffin-embedded CCA tissue samples were collected from CCA patients who had undergone surgery at Srinagarind Hospital, Khon Kaen University, from February 2007 to December 2016. These samples were kept by the Cholangiocarcinoma Research Institute (CARI), Faculty of Medicine, Khon Kaen University, under the ethics approval number HE571283. CCA cell lines, including KKU023, KKU055, KKU100, KKU156 and KKU213, were used for this study. The KKU023 cell line was established from the proven bile duct cancer of a patient living in the northeast region of Thailand with written consent from the patient. The KKU055, KKU100, KKU156 and KKU213 cell lines were obtained from the Japanese Collection of Research Bioresources (JCRB) Cell Bank, Osaka, Japan. These cells were cultured in Ham’s F12 media supplemented with inactivated 10% fetal bovine serum (FBS) and 100 U/ml of penicillin-streptomycin, at 37°C with 5% CO_2_.

### Chemicals and Reagents

Monoclonal antibody against ACC (catalog Number: ab45174) and polyclonal antibody against FASN (catalog Number: ab22759) were purchased from Abcam, United Kingdom. Monoclonal antibody against HMGCR (catalog Number: SAB4200528) was purchased from Sigma-Aldrich, United States. Short hairpin RNA (shRNA) was purchased from Sigma-Aldrich, United States. The Annexin V/PI staining kit was purchased from Invitrogen, United States. High-performance liquid chromatography (HPLC)-grade methanol, chloroform and water were purchased from Merck, Germany.

### Immunohistochemistry and Grading System

Immunohistochemical staining was performed in order to investigate lipid-metabolizing enzyme expression in human CCA tissues. Paraffin-embedded tissues were de-paraffinized and rehydrated with xylene followed by 100, 90, 80 and 70% ethanol. Antigen retrieval was performed by microwave cooking with 10 mM sodium citrate buffer for 10 min. The tissue sections were treated for 30 min with 0.3% hydrogen peroxide to block the activity of endogenous hydrogen peroxide and 10% skim milk to block the non-specific binding. The tissue sections were incubated with primary antibodies at room temperature for 1 h followed by 4°C overnight, then incubated with secondary antibodies conjugated with horseradish peroxidase (HRP) for 3 h. The signal was developed using a 3,3′-diaminobenzidine tetrahydrochloride (DAB) substrate kit (Vector Laboratories, United States) for 5–10 min. Tissue sections were counterstained with Mayer’s haematoxylin for 5 min and dehydrated with 70, 80, 90, 100% ethanol and xylene then mounted with permount. The stained tissue sections were observed under a microscope. The lipid-metabolizing enzyme expression was analyzed according to the staining frequency and intensity. The staining frequency of enzymes was semi-qualitatively scored based on the positive cells percentage, 0% = negative, 1–25% = +1, 26–50% = +2 and >50% = +3. The staining intensity of enzymes was scored as weak = 1, moderate = 2 and strong = 3. The lipid-metabolizing enzyme expression was divided into low or high expression group using median as the cut-off.

### Western Blot Analysis

The cell pelltes were extracted with NP40 lysis buffer containing a cocktail of protease and phosphatase inhibitors. Protein concentration was determined using the Pierce BCA™ Protein Assay kit (Thermo Fisher Scientific, United States). Protein extracts were solubilized in sample buffer containing sodium dodecyl sulfate (SDS) and β-mercaptoethanol and boiled at 95°C for 5 min. Protein extracts were separated by 8% sodium dodecyl sulfate polyacrylamide gel electrophoresis (SDS-PAGE), transferred onto PVDF membranes and blocked by 5% skimmed milk for 1 h at room temperature. The membranes were probed with specific primary antibodies for 1 h at room temperature, incubated at 4°C with gentle shaking overnight, then probed with secondary antibodies. The membranes were exposed to ECL™ Prime Western Blotting Detection Reagent for chemiluminescent detection (GE Healthcare, United States). The band density on the membranes were quantified by ImageQuant™ Imager (GE Healthcare, United States). In this study, β-actin antibody (Sigma Aldrich, United States) was used as an internal loading control.

### Fatty Acid Synthase Gene Knockdown

CCA cell lines (KKU055 and KKU213) were plated into 48-well plates. After 24 h, 100 µl of concentrated lentivirus was added to the medium containing 8 µg/ml polybrene. Two shRNAs (sh1 and sh2) targeting FASN were used, and control shRNA was used for the control cells. Cells were incubated at 37°C in a humidified atmosphere with 5% CO_2_. Fresh medium was replaced 24 h post-transduction. FASN knockdown cells were selected with medium containing puromycin. The efficiency of shRNA transfection was determined using western blot analysis.

### Cell Proliferation Assay

A sulforhodamine B (SRB) assay was used to determine the cell proliferation. The FASN-knockdown CCA cell lines were plated in triplicate in 96-well plates and incubated for 24, 48, and 72 h. Then, the cells were fixed with 10% trichloroacetic acid (TCA) at 4°C for 1 h and stained with 0.4% SRB for 30 min. The protein-bound stained cells were dissolved with 10 mM tris-base, pH 10.5 for 1 h on shaking plate. The absorbance was measured at 540 nm by microplate reader (TECAN Trading, Switzerland).

### Cell Migration Assay

A wound-healing assay was used to evaluate the cell migration. The FASN-knockdown CCA cell lines were cultured in 24-well plates and incubated at 37°C in a humidified atmosphere with 5% CO_2_ until the cells became more than 90% confluent. Cell monolayers were scratched using a sterile tip and then washed several times with 1X PBS to remove the cell debris. Cell migration in the wounded area was observed every 6 h and photographed under a microscope.

### Cell Invasion Assay

A Boyden chamber assay was used to perform the cell invasion assay. The complete medium was added to the lower chamber while serum-free medium was added to the upper chamber. The FASN knockdown CCA cell lines were cultured in the upper chamber and incubated at 37°C in a humidified atmosphere with 5% CO_2_ for 24 h. The cells attached to the filter were fixed with methanol for 30 min at room temperature and stained with hematoxylin overnight. After that, the filter was dried at 60°C for 30 min and mounted with permount, then observed under a microscope.

### Cell Cycle Assay

The FASN knockdown CCA cell lines were plated into 6-well plates and incubated at 37°C in a humidified atmosphere with 5% CO_2_ for 72 h. The cells were fixed with 70% ethanol and incubated at 4°C overnight. PI/RNase staining buffer was added to the fixed cells followed by incubation at 4°C in the dark for 30 min. The stained cells were detected using a flow cytometer (BD Bioscience, United States).

### Apoptosis Assay

Apoptosis was detected using the Annexin V/PI staining kit. The FASN knockdown CCA cell lines were seeded into 6-well plates and incubated at 37°C in a humidified atmosphere with 5% CO_2_ for 72 h. The cells were collected, washed with 1X PBS and resuspended in binding buffer, then annexin V and propidium iodide (PI) were added and incubated at room temperature for 15 min. The stained cells were measured using a flow cytometer (BD Bioscience, United States).

### Liquid Chromatography-Mass Spectrometry-Based Metabolomics

Global metabolic profiles were acquired using LC-MS in the cell pellets from FASN knockdown CCA cell lines. The cells were quenched in ice-cold methanol and snap-frozen in liquid nitrogen, then sonicated (3 cycles of pulse at 30 s, off 5 s, at amplitude of 40). Water (HPLC grade) and chloroform were added into the samples for dual-phase extraction followed by centrifugation at 4,000 g, 4°C for 20 min. Then, 450 μl of aqueous phase was collected into a microcentrifuge tube. The solvent contained in the aqueous phase was removed using speed vacuum concentrator (Labconco, United states). The aqueous phase sample was reconstituted in reconstitution buffer and transferred into a glass vial for LC-MS data acquisition. In addition, the samples were analyzed using a reverse-phase (RP) liquid chromatography platform. The separated part was analysed using the ultra-high performance liquid chromatography (UHPLC) system. A C18 column (2.1 × 100 mm, 2 μm) (Bruker, Germany) was used with the column temperature set at 40°C. Mobile phase A comprised water 100% mixed with 0.1% formic acid, and mobile phase B comprised acetonitrile 100% mixed with 0.1% formic acid. The elution gradient was set at a flow rate of 0.4 ml/min. Sodium formate (2 mM) was used as the calibrant. Mass spectrometry was performed on a compact electrospray ionization-quadrupole time-of-flight (ESI-Q-TOF) system (Bruker, Germany). The blank and quality control (QC) samples were randomly analyzed to reduce instrumentation artifacts.

### Liquid Chromatography-Mass Spectrometry/MS Data Processing and Metabolite Identification

Following data acquisition, MetaboScape 4.0 (Bruker, Germany) software was used for feature extraction. The data file was subjected to MetaboAnalyst 5.0 (University of Alberta, Canada) software for statistical analysis (Pang et al., 2020). The Mann-Whitney *U*-test was used, and only features with an *p*-value less than 0.05 were selected for metabolite identification. The metabolite identification was performed by matching the m/z with online databases (Human Metabolome Database; HMDB and METLIN). Additionally, the fragmentation patterns of each feature were also investigated. The level of assignment was classified based on the previously published criteria (Vorkas et al., 2015) which were: 1) m/z matched to database, 2) m/z matched to database and fragmentation pattern matched to in silico fragmentation pattern, 3) fragmentation pattern matched to database or literature, 4) retention-time matched to standard compound, 5) fragmentation pattern matched to standard compound.

### Statistical Analysis

The association between lipid-metabolizing enzyme expression with the clinicopathological characteristics of CCA patients was analzed using chi-square test. Survival analysis was performed by Kaplan-Meier (log-rank) analysis (Statistical Package for the Social Sciences; SPSS software v.19), statistical significance was considered if the *p*-value was less than 0.05. For functional analysis, the significant differences were determined using unpaired *t*-test (*p*-value < 0.05) on GraphPad Prism 5 (California, United states) software.

## Results

### Correlation of Lipid-Metabolizing Enzyme Expression With Clinicopathological Features of Cholangiocarcinoma Patients

**FIGURE 1:**
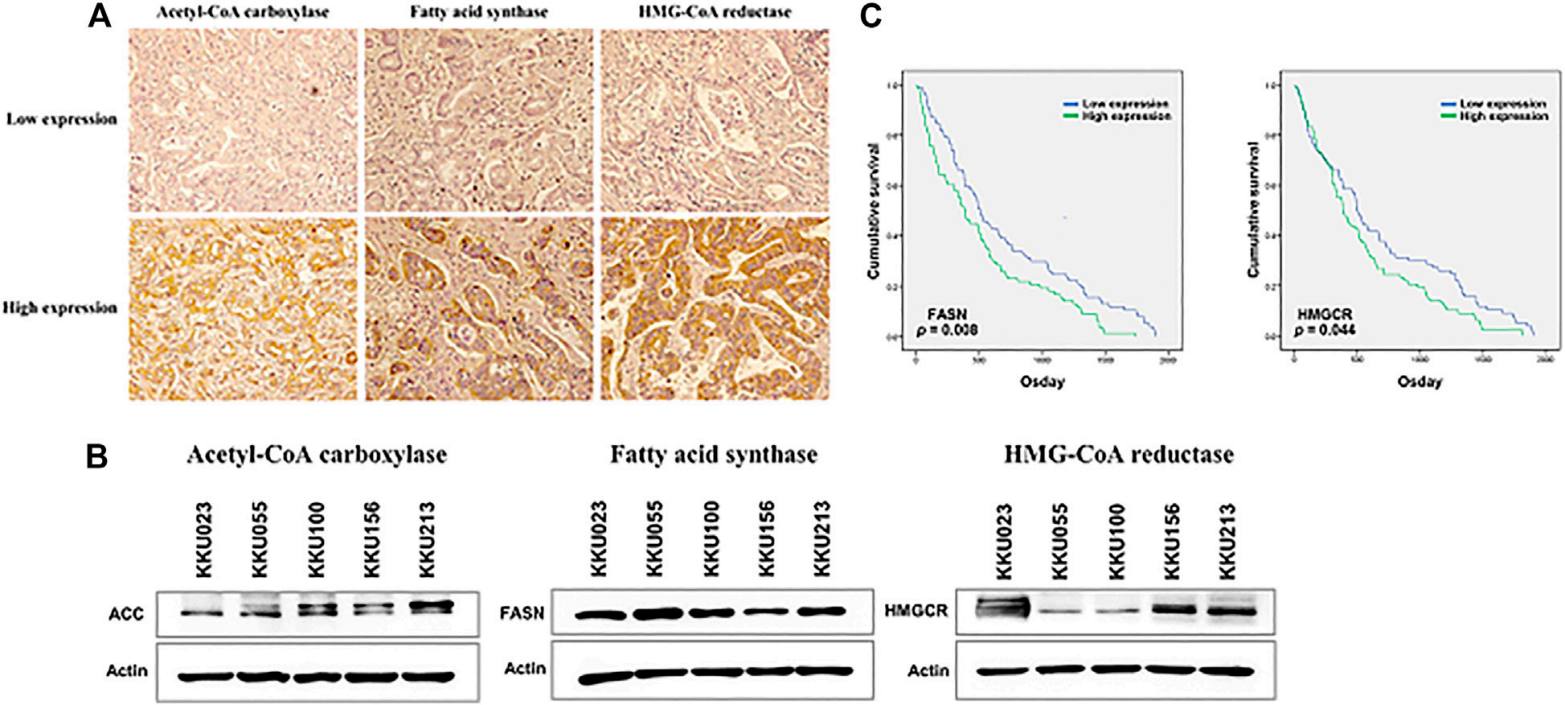
The expression of lipid-metabolizing enzyme in CCA tissues and cell lines. **(A)** Immunohistochemical staining of ACC, FASN and HMGCR using a human CCA tissues microarray; low expression is shown in the upper panel and high expression in the lower panel. **(B)** ACC, FASN and HMGCR protein expression in five CCA cell lines using western blot analysis. **(C)** The Kaplan-Meier survival curves of CCA patients were examined according to lipid-metabolizing enzymes expression; the *p*-values were calculated by the log-rank test.

The expression levels of the lipid-metabolizing enzymes were investigated using immunohistochemical staining. The low and high expression of lipid-metabolizing enzymes, including ACC, FASN and HMGCR in human CCA tissues are represented in Figure 1A. To investigate the correlation between ACC, FASN and HMGCR expression and clinicopathological features of CCA patients, a total of 155 CCA patients were studied; 64 cases (41%) were females and 91 cases (59%) males. The ages ranged between 39 and 82 years (median = 61 years). Ninety seven cases (63%) were classified as iCCA while 58 cases (37%) were extrahepatic CCA (pCCA or dCCA). The histology typing resulted in 76 cases (49%) of the papillary type and 79 cases (51%) of other types. Fifty seven cases (37%) were classified as primary tumor (T) stage I or II whereas 98 cases (63%) were T stage III or IV. Among 155 patients, 64 (41%) had regional lymph node metastasis (N), and only 7 cases (5%) presented with distant metastases (M). In this study, 60 cases (39%) were divided into early stage (TNM stage I, II) while 95 cases (61%) were separated into late or advanced stage (TNM stage III, IV) and recurrence was detected after surgery in 60 cases (39%). The results demonstrated that high expression of FASN significantly correlated with advanced stage in CCA patients (*p* = 0.041, Table 1). Moreover, western blot analysis showed that ACC, FASN and HMGCR expression was observable in the five CCA cell lines - KKU023, KKU055, KKU100, KKU156 and KKU213 (Figure 1B).

**TABLE 1:** The correlation of lipid-metabolizing enzyme expression with the clinicopathological features of CCA patients.

Variable	ACC		*p*	FASN		*p*	HMGCR		*p*
	Low	High		Low	High		Low	High	
**Sex**			0.216			0.295			0.170
Female	28 (43.8)	36 (56.2)		35 (54.7)	29 (45.3)		36 (56.3)	28 (43.7)	
Male	49 (53.8)	42 (46.2)		42 (46.2)	49 (53.8)		41 (45.1)	50 (54.9)	
**Age (year)**			0.576			0.127			0.576
<61	39 (52.0)	36 (48.0)		42 (56.0)	33 (44.0)		39 (52.0)	36 (48.0)	
≥61	38 (47.5)	42 (52.5)		35 (43.8)	45 (56.2)		38 (47.5)	42 (52.5)	
**Tumor location**			0.950			0.085			0.206
Intrahepatic	48 (49.5)	49 (50.5)		43 (44.3)	54 (55.7)		52 (53.6)	45 (46.4)	
Extrahepatic	29 (50.0)	29 (50.0)		34 (58.6)	24 (41.4)		25 (43.1)	33 (56.9)	
**Histology**			0.689			0.471			0.573
Papillary	39 (51.3)	37 (48.7)		40 (52.6)	36 (47.4)		36 (47.4)	40 (52.6)	
Others	38 (48.1)	41 (51.9)		37 (46.8)	42 (53.2)		41 (51.9)	38 (48.1)	
**Primary tumor (T)**			0.440			0.220			0.440
I, II	26 (45.6)	31 (54.4)		32 (56.1)	25 (43.9)		26 (45.6)	31 (54.4)	
III, IV	51 (52.0)	47 (48.0)		45 (45.9)	53 (54.1)		51 (52.0)	47 (48.0)	
**Lymph nodes metastasis (N)**			0.694			0.796			0.694
No	44 (48.4)	47 (51.6)		46 (50.5)	45 (49.5)		44 (48.4)	47 (51.6)	
Yes	33 (51.6)	31 (48.4)		31 (48.4)	33 (51.6)		33 (51.6)	31 (48.4)	
**Distant metastasis (M)**			0.686			0.712			0.051
No	73 (49.3)	75 (50.7)		74 (50.0)	74 (50.0)		71 (48.0)	77 (52.0)	
Yes	4 (57.1)	3 (42.9)		3 (42.9)	4 (57.1)		6 (85.7)	1 (14.3)	
**TNM stage**			0.694			**0.041**			0.790
I, II	31 (51.7)	29 (48.3)		36 (60.0)	24 (40.0)		29 (48.3)	31 (51.7)	
III, IV	46 (48.4)	49 (51.6)		41 (43.2)	54 (56.8)		48 (50.5)	47 (49.5)	
**Recurrence**			0.209			0.167			0.949
No	51 (53.7)	44 (46.3)		43 (45.3)	52 (54.7)		47 (49.5)	48 (50.5)	
Yes	26 (43.3)	34 (56.7)		34 (56.7)	26 (43.3)		30 (50.0)	30 (50.0)	

ACC, Acetyl-CoA carboxylase; FASN, Fatty acid synthase; HMGCR, HMG-CoA reductase; Low, Low expression; High, High expression; TNM stage, Size of primary tumor-node metastasis-distant metastasis.

### Kaplan-Meier Survival Analysis of Lipid-Metabolizing Enzyme Expression in Cholangiocarcinoma Patients

To evaluate the prognostic role of lipid-metabolizing enzyme expression in CCA patients, the Kaplan-Meier survival curves showed that CCA patients with high expression of FASN and HMGCR were associated with shorter overall survival times (*p* = 0.008 and *p* = 0.044, respectively) (Figure 1C).

### Western Blot Analysis of Fatty Acid Synthase Knockdown on Cholangiocarcinoma Cells

**FIGURE 2:**
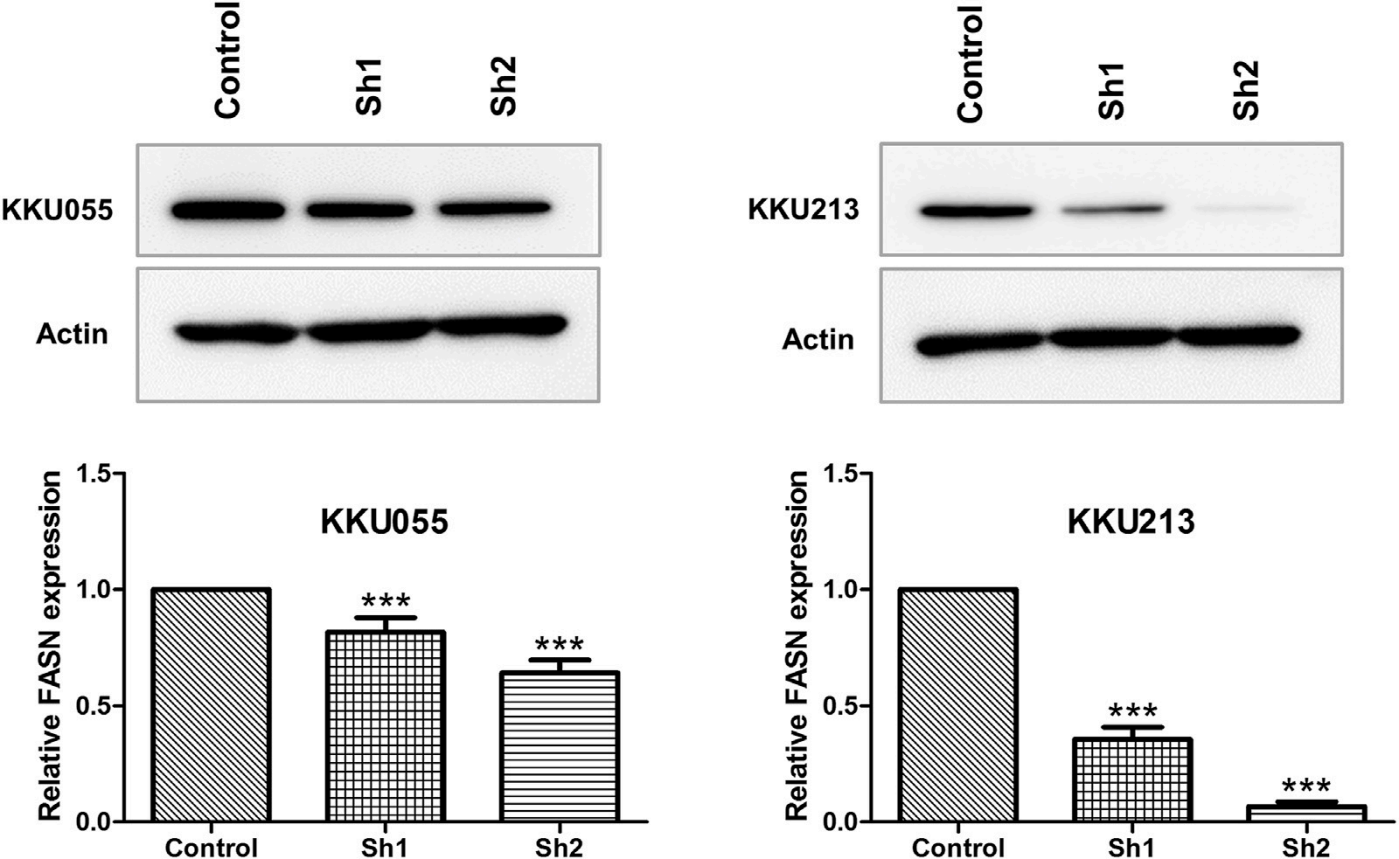
Western blot analysis of FASN knockdown efficiency in CCA cell lines. The protein expressions of FASN in KKU055 and KKU213 cells stably transfected with control shRNA (control) or shRNA against FASN (sh1 and sh2) are shown at the top. The bands were quantified and shown at the bottom. The data are presented as the mean  ± standard deviation (SD) of three independent experiments. Statistical significance was determined as ***p < 0.001.

To examine the roles of FASN in CCA cell lines, lentiviral transduction was used to stably knockdown FASN expression in KKU055 and KKU213 cell lines. Western blot analysis was performed to ensure the knockdown efficiency; the expression of FASN was lowered at protein levels in cells transfected with shRNA against FASN (sh1 and sh2) (*p* < 0.001) compared with the control counterpart (Figure 2).

### Effect of Fatty Acid Synthase Knockdown on Cholangiocarcinoma Cell Growth

**FIGURE 3:**
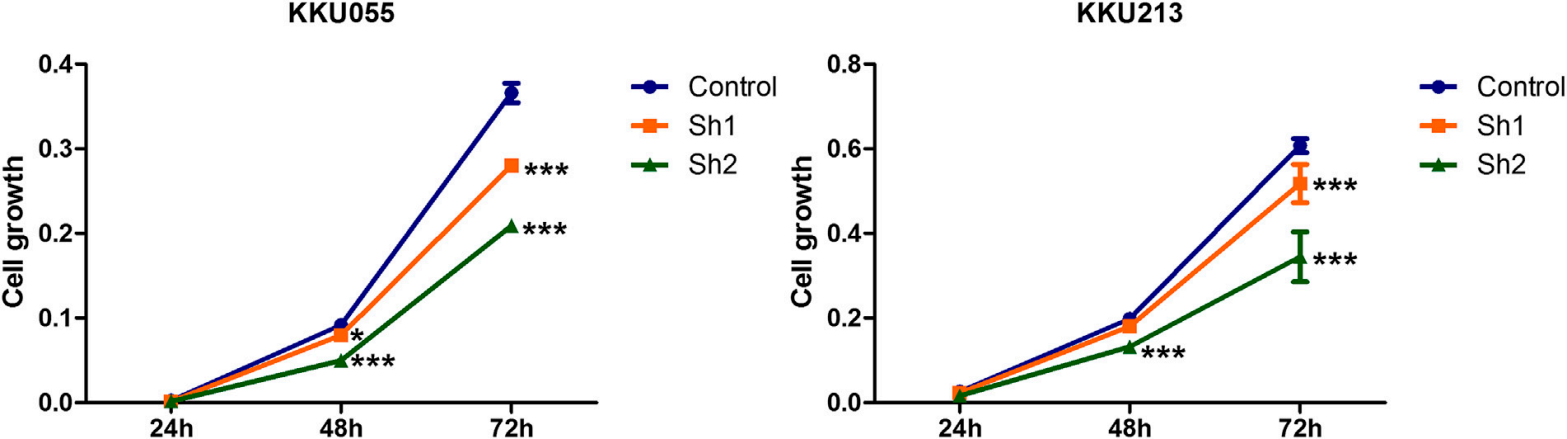
The effect of FASN knockdown on CCA cell growth. The SRB assay was carried out for KKU055 and KKU213 cells at 24, 48 and 72 h post-transfection with shRNA targeting FASN. The data are presented as the mean  ± standard deviation (SD) of three independent experiments. Statistical significance was determined as *p < 0.05 and ***p < 0.001, respectively.

SRB assay was used to explore the effect of FASN knockdown on CCA cell growth (KKU055 and KKU213 cell lines) at 24, 48 and 72 h post-transfection with shRNA targeting FASN. The SRB assay showed a decrease in CCA cell growth in a time-dependent manner, resulting in a significant decrease in the sh1 group at 48 h (*p* < 0.05) and 72 h (*p* < 0.001), and in the sh2 group at 48 and 72 h (*p* < 0.001) in KKU055 cells compared with the control cells. Similarly, for the KKU213 cells, the cell growth was significantly decreased in the sh1 group at 72 h (*p* < 0.001), and in the sh2 group at 48 and 72 h (*p* < 0.001) (Figure 3).

### Effect of Fatty Acid Synthase Knockdown on Cholangiocarcinoma Cell Migration

**FIGURE 4:**
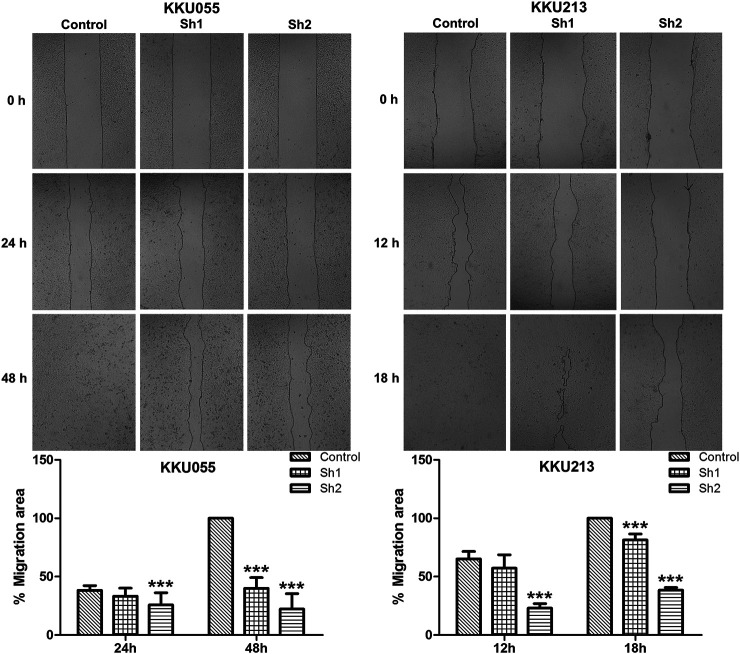
The effect of FASN knockdown on CCA cell migration. The wound-healing assay was conducted on KKU055 and KKU213 cells as shown in the upper panels, respectively. The migration areas were calculated as shown in the lower graphs. The data are presented as the mean  ± standard deviation (SD) of three independent experiments. Statistical significance was determined as ***p < 0.001.

The wound-healing assay revealed that FASN knockdown in KKU055 and KKU213 significantly reduced their cell migration ability. Both the sh1 and sh2 groups showed significant reduction at 48 h (*p* < 0.001) in KKU055 cells compared with the control cells. For the KKU213 cells, the migration ability of the sh1 and sh2 groups were significantly reduced at 18 h (*p* < 0.001) (Figure 4).

### Effect of Fatty Acid Synthase Knockdown on Cholangiocarcinoma Cell Invasion

**FIGURE 5:**
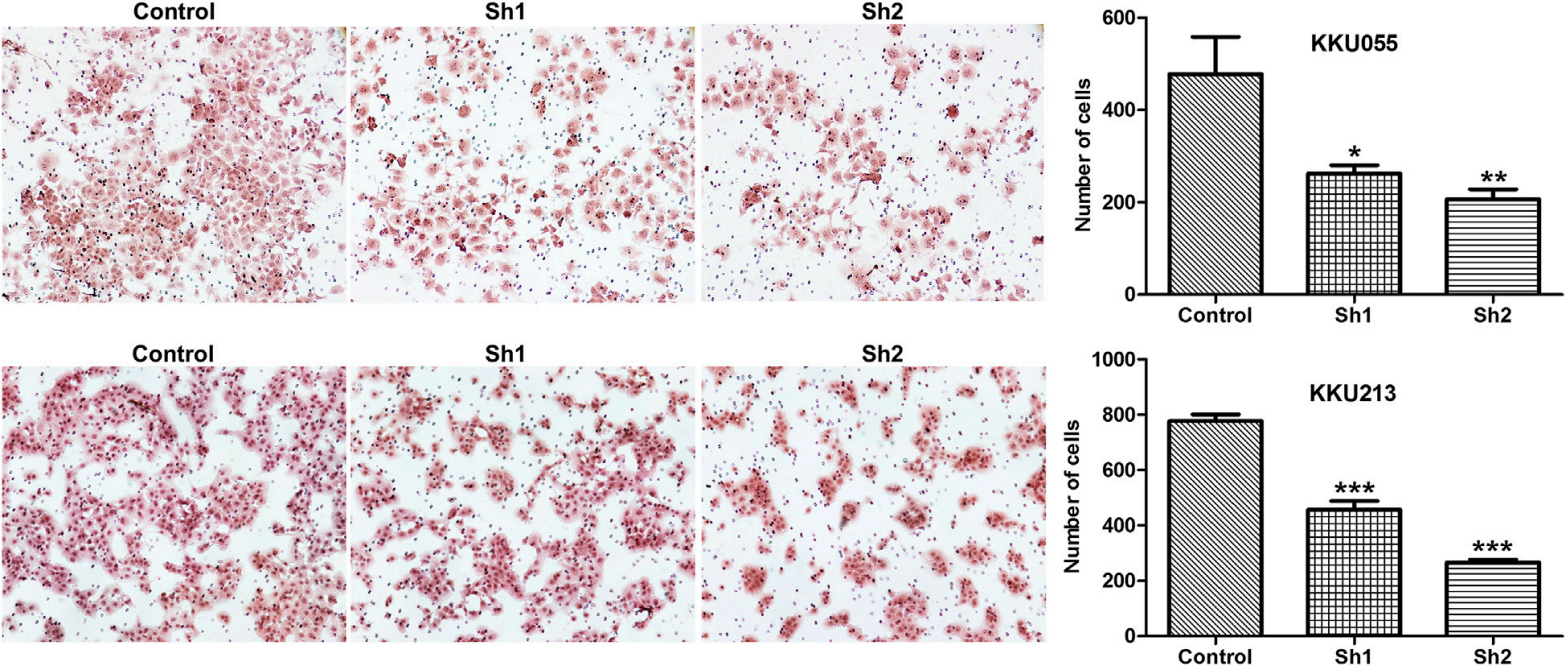
The effect of FASN knockdown on CCA cell invasion. A Boyden chamber assay was evaluated in KKU055 and KKU213 cells as revealed on the left. The number of cells were counted as indicated on the right. The data are presented as the mean  ± standard deviation (SD) of three independent experiments. Statistical significance was determined as *p < 0.05, **p < 0.01 and ***p < 0.001, respectively.

The Boyden chamber assay was used to study the effect of FASN knockdown on CCA cell invasion (KKU055 and KKU213 cell lines). The assay showed that FASN knockdown led to a significant reduction in the number of cells in the sh1 group (*p* < 0.05) and the sh2 group (*p* < 0.01) in KKU055 cells compared with the control cells. Similarly, FASN knockdown significantly reduced the number of cells in both sh1 and sh2 groups (*p* < 0.001) in KKU213 cells (Figure 5).

### Effect of Fatty Acid Synthase Knockdown on the Cell Cycle of Cholangiocarcinoma Cells

**FIGURE 6:**
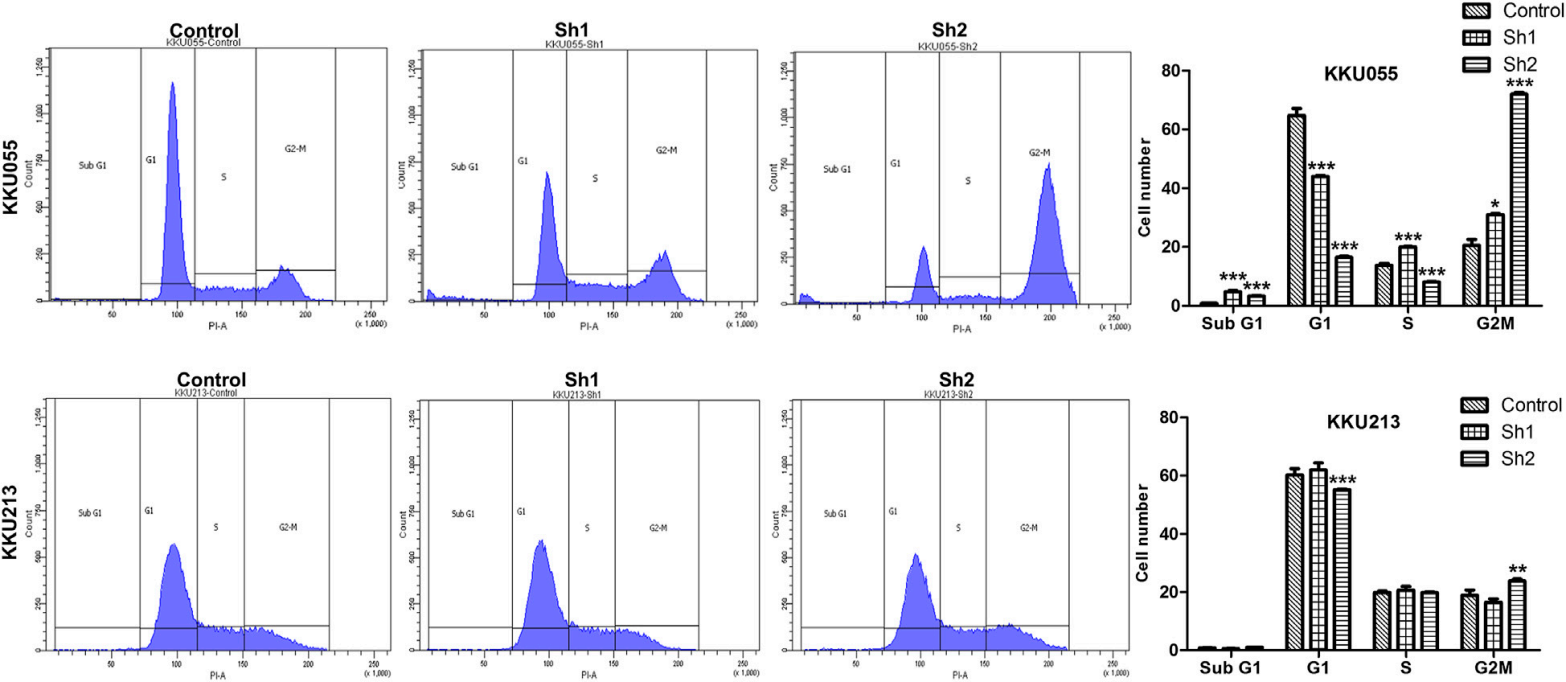
The effect of FASN knockdown on the CCA cell cycle of KKU055 and KKU213 cells. The percentage of cells in each phase as measured by flow cytometry with PI staining is shown on the left. The cell numbers were calculated as shown on the right. The data are presented as the mean  ± standard deviation (SD) of three independent experiments. Statistical significance was determined as *p < 0.05, **p < 0.01 and ***p < 0.001.

Effect of FASN knockdown on the cell cycle was analyzed using flow cytometry with propidium iodide (PI) DNA staining to determine the cell cycle distribution of KKU055 and KKU213 cells. For KKU055 cells, the FASN knockdown yielded significantly decreased proportion of cells in the G1 phase of the sh1 and sh2 groups (*p* < 0.001) and a significant increase of cells in the G2M phase of the sh1 (*p* < 0.05) and sh2 (*p* < 0.001) groups compared with the control cells. For KKU213 cells, the proportion of cells in the G1 phase was significantly decreased, and G2M phase was elevated in the sh2 group (*p* < 0.001 and *p* < 0.01, respectively) (Figure 6).

### Effect of Fatty Acid Synthase Knockdown on Apoptosis of Cholangiocarcinoma Cells

**FIGURE 7:**
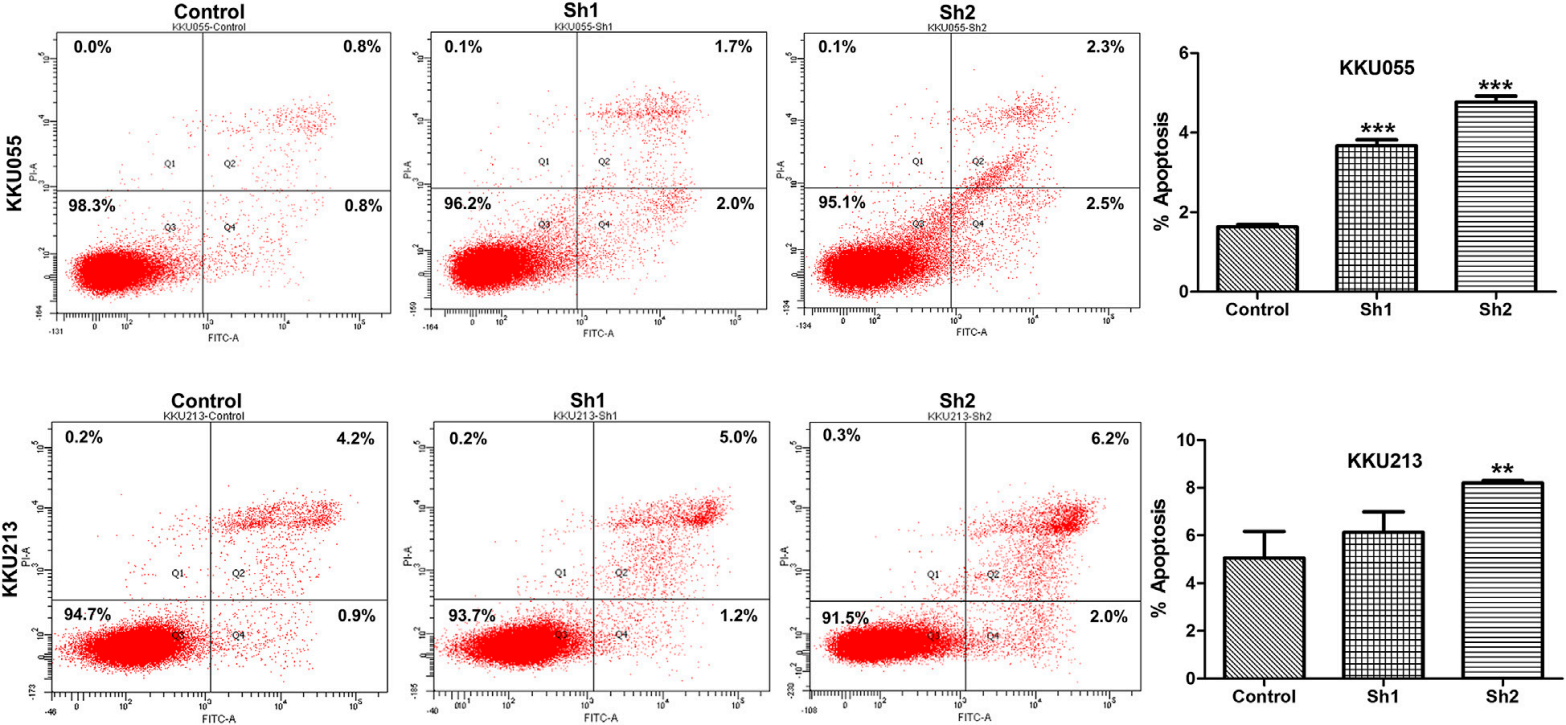
The effect of FASN knockdown on cell apoptosis based on quantitative flow cytometry measurements in KKU055 and KKU213 cells. Apoptotic cells were detected by annexin V/PI staining as shown on the left. The percentages are shown on the right. The data are presented as the mean  ± standard deviation (SD) of three independent experiments. Statistical significance was determined as **p < 0.01 and ***p < 0.001.

To investigate the effect of FASN knockdown on the apoptosis of CCA cells, flow cytometry with annexin V/PI staining was used to confirm programmed cell death in KKU055 and KKU213 cells. Knockdown of FASN expression significantly increased the number of apoptotic cells in the sh1 and sh2 groups (*p* < 0.001) for KKU055 cells, as well as significantly increased the apoptotic cells in the sh2 group (*p* < 0.01) for KKU213 cells compared with the control cells (Figure 7).

### Metabolic Profiles of Fatty Acid Synthase Knockdown Cholangiocarcinoma Cells

**FIGURE 8:**
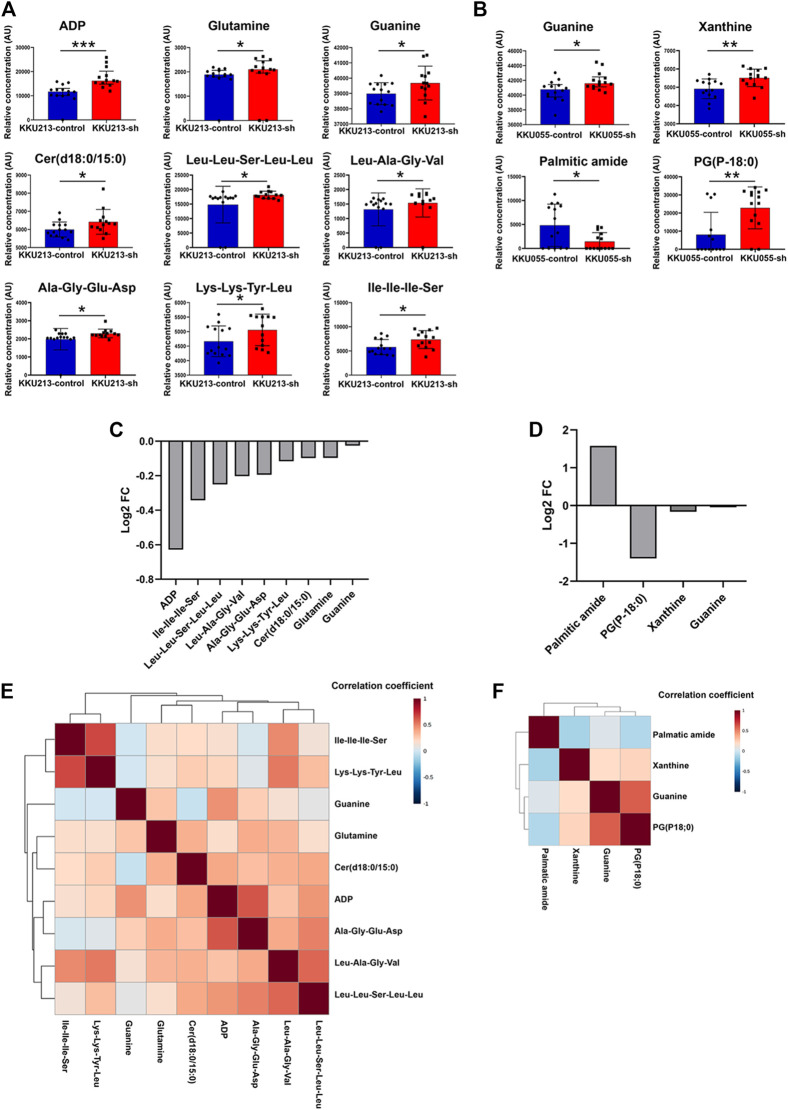
The metabolic profiles of FASN knockdown CCA cells. **(A)** Relative concentrations of candidate metabolites in KKU213, and **(B)** in KKU055 cells; blue and red bar graphs indicate the control and FASN knockdown groups, respectively. Error bars represent the standard deviation (SD) of samples. **(C)** Log_2_ of fold change (FC) of metabolites comparing the control and FASN knockdown groups in KKU213, and **(D)** in KKU055 cells. **(E)** Heatmap analysis with hierarchical clustering of all significant metabolites in KKU213, and **(F)** in KKU055 cells. The correlation coefficient from Spearman correlation is indicated in each colored cell on the map. The scale code is shown on the right (red and blue colors indicate positive and negative correlations, respectively). Statistical significance was determined as *p < 0.05, **p < 0.01 and ***p < 0.001, respectively.

**FIGURE 9:**
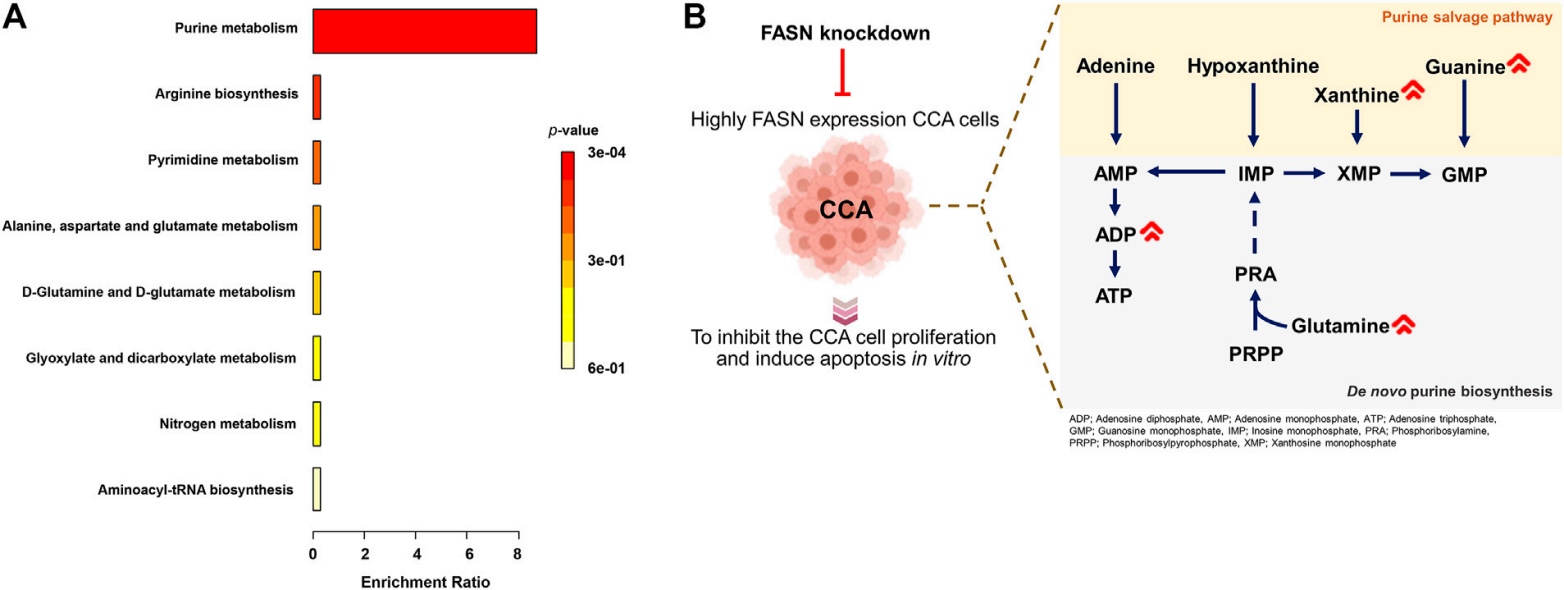
Metabolic networks involved in FASN knockdown. **(A)** Enrichment analysis on identified metabolites from KKU213 cells. **(B)** A schematic diagram of the major metabolic networks involved in FASN knockdown.

The above results showed that FASN knockdown can inhibit the progression of CCA cells. In order to explore the mechanisms underlying such effects, a metabolomics study was performed on cells transfected with control shRNA (control) and cells stably transfected with shRNA against FASN (FASN knockdown; sh2) in both KKU055 and KKU213 cells. Adenosine diphosphate (ADP), glutamine, guanine, Cer(d18:0/15:0) and peptides were significantly different between the control and FASN knockdown in KKU213 cells (Figure 8A). Guanine, xanthine, palmitic amide and PG (P-18:0) showed a significant difference in levels between the control and FASN knockdown KKU055 cells (Figure 8B). Moreover, the fold-change (FC) of each metabolite was examined. The result showed that ADP significantly increased in the FASN knockdown KKU213 cell line with an FC more than 1.5 (Figure 8C). Furthermore, PG (P-18:0) significantly increased in the FASN knockdown KKU055 cell line while palmitic amide significantly decreased in the FASN knockdown KKU055 cell line with an FC more than 1.5 (Figure 8D). In addition, a heatmap with hierarchical clustering was performed for selected metabolites, the results were shown in Figures 8E,F. To explore the relevant pathways to the differential metabolites affected by FASN knockdown, enrichment analysis was performed using MetaboAnalyst 5.0 software. The result on KKU213 showed that purine metabolism was the most relevant pathway affected by the FASN knockdown (adjusted *p*-value < 0.05) (Figure 9A). A schematic diagram of the metabolic networks involved in FASN knockdown is depicted in Figure 9B.

## Discussion

It is commonly recognized that metabolic reprogramming is a hallmark of cancer, and cancer cells require certain changes in metabolism to assist their unrestricted proliferation and metastasis (Hanahan & Weinberg, 2011). Among all the metabolic shifts, activation of the *de novo* lipid biosynthesis pathway is essential for carcinogenesis. There is evidence that an up-regulation of lipid biosynthesis enzymes, including ACC, FASN and HMGCR, can be found in many cancer types. ACC is a major regulator of fatty acid metabolism that is involved in the conversion of acetyl-CoA into malonyl-CoA which is a critical substrate for fatty acid synthesis. Compared with normal cells, cancer cells have a higher synthesis of fatty acid. The factors involved in lipid synthesis are also detected in cell proliferation and viability of certain cancers. In non-small-cell lung cancer, ACC inhibition reduces *de novo* lipid synthesis and decreases cell growth and viability (Svensson et al., 2017). In human U87 EGFRvIII, ACC knockdown not only inhibits *de novo* lipogenesis, but also diminishes U87 EGFRvIII cellular proliferation and viability (Jones et al., 2017). In liver cancer, ACC overexpression is correlated with a poorer prognosis and shorter survival time in liver cancer patients. Moreover, ACC knockdown resulted in decreased liver cancer cell growth and migration. Additionally, ACC knockdown also decreases the mRNA and protein expression levels of the cell proliferation-associated genes; MYCN, JUN, cyclin D1 and cyclin A2 (Ye et al., 2019).

FASN, a crucial enzyme in *de novo* fatty acid synthesis, has been found overexpressed in cancers (Sun et al., 2018; Zielinska et al., 2018). Furthermore, studies showed that the overexpression of FASN is associated with cancer cell proliferation, metastasis, poor prognosis and a high risk of recurrence (Visca et al., 2000; Lu et al., 2019). In addition, FASN overexpression is correlated with lymph node metastasis, TNM stage and a poor prognosis in colorectal cancer patients. FASN knockdown resulted in reduced colorectal cancer cell proliferation and migration, while FASN overexpression exhibited the reverse phenomenon. Moreover, a potential mechanism through FASN-induced colorectal cancer proliferation and metastasis upon its regulation of the AMPK/mTOR pathway by increasing ATP production, resulted in inhibition of AMPK and activation of mTOR has been demostrated (Lu et al., 2019).

HMGCR, the target of statin, is the rate-limiting enzyme for the *in vivo* cholesterol synthesis (Sharpe & Brown, 2013). HMGCR is up-regulated in the gastric cancer tissues that was evident in the previous clinical study (Chushi et al., 2016). HMGCR overexpression promotes the growth and migration of gastric cancer cells, while HMGCR knockdown has the opposite effects both *in vitro* and *in vivo*. In addition, HMGCR activates Hedgehog/Gli1 signaling and promotes the expression of Gli1 target genes. Statin combined with the small molecular inhibitor for Hedgehog signaling might be effective for the gastric cancer treatment (Chushi et al., 2016). HMGCR has been involved in the malignant transformation of normal breast cancer cells, and also in the early-stage tumorigenesis (Singh et al., 2015). Additionally, HMGCR has been reported to foster the growth and migration of the glioma cells (Qiu et al., 2015).

These data demonstrated that ACC, FASN and HMGCR are promising potential targets for cancer treatment. Therefore, the elucidation of changes in lipid metabolism in cancer is required to develop therapeutic targets for various human cancers. In the present study, lipid-metabolizing enzyme expression was higher in CCA tissues. Moreover, clinical data analyses demonstrated the significant association of high FASN expression with advanced-stage CCA. In addition, survival analysis showed that a high expression of FASN and HMGCR were correlated with a shorter survival for CCA patients. We also demonstrated that silencing FASN expression significantly inhibited CCA cell growth, migration, invasion, cell cycle and induced apoptosis *in vitro*.

Our findings reveal metabolic alterations in CCA cells in response to FASN knockdown, determined through an untargeted metabolomics analysis using the UHPLC-MS/MS technique. It demonstrates that global metabolomics is beneficial for study of cellular metabolism. Purines, one of the most abundant metabolic products, are vital biological components that provide the building blocks (adenine and guanine) of DNA and RNA (Pedley and Benkovic, 2018). Purines are also considered key components of several essential biomolecules including ATP, GTP, cAMP, NADH and coenzyme A. These biomolecules are involved in several pathways of biological machinery such as energy production, cellular signaling pathways, redox metabolism and fatty acid synthesis (Virgilio & Adinol, 2017). There are two main purine biosynthesis pathways in mammalian cells, namely the complementary salvage pathway and the purine *de novo* biosynthesis pathway (Yin et al., 2018). Rapidly proliferating cells and tumor cells require higher amount of purines, which are synthesized through the up-regulation of the purine *de novo* biosynthesis pathway (Su et al., 2021). Under normal physiological conditions, most of the cellular requirements for purine from the recycling of degraded bases *via* the salvage pathway. The salvage process uses hypoxanthine-guanine phosphoribosyl transferase (HPRT) to convert hypoxanthine and guanine to inosine monophosphate (IMP) and guanosine monophosphate (GMP), respectively. Adenine can also be combined with phosphoribosyl pyrophosphate (PRPP) to generate adenosine monophosphate (AMP) in a process catalyzed by adenine phosphoribosyl transferase (APRT) (Pedley and Benkovic, 2018). Under cellular conditions requiring higher purine levels, the intracellular purine demand by upregulating the *de novo* biosynthetic pathway, is a highly conserved, energy intensive pathway that generates IMP from PRPP. Purine nucleotide synthesis begins with PRPP and leads to the first fully formed nucleotide: IMP. Through a series of reactions utilizing ATP, tetrahydrofolate derivatives, glutamine, glycine and aspartate, this pathway yields IMP, which signifies a branch point for purine biosynthesis because of its conversion into either AMP or GMP through two distinct reaction pathways (Tolstikov et al., 2014).

Our findings collectively suggest the significant FASN knockdown-associated metabolic changes in purine metabolism of CCA cells that is in agreement with the previously reported mechanism of FASN knockdown DNA-targeting. Purine metabolism is essential for the production of DNA components required for CCA cell proliferation, and its inhibition can lead to apoptosis. In the present study, ADP, glutamine and guanine showed a significant difference between the control and FASN knockdown in KKU213 cells. ADP level decreased in the control group accompanied with lower levels of glutamine and guanine while FASN knockdown cells demonstrated the elevated level of ADP indicating the suppressed ATP content. Similarly, levels of guanine and xanthine were found to be significantly different between the control and FASN knockdown in KKU055 cells as can be evident by decreased levels of guanine and xanthine in the control group. In addition, purine metabolism is the most relevant pathway involved in FASN knockdown. Glutamine, guanine and xanthine can be used to sustain high rates of cellular proliferation as a key nitrogen donor in purine nucleotide biosynthesis, and also serves as an essential substrate for key enzymes involved in the synthesis of purine nucleotides. The mechanism of FASN knockdown as a suppressor of purine metabolism leads to the inhibition of ATP production and less utilization of purine nucleotides substrate for DNA synthesis compared with control group, which in turn leads to the inhibition of CCA cell proliferation and induces apoptosis.

Based on our results, the two CCA cell lines behave differently with FASN knockdown, which reflect the fact that CCA is a heterogeneous group of malignancies based on histological and molecular characterization (Kendall et al., 2019). Moreover, CCA can emerge at different sites of the biliary tree and with different macroscopic or morphological features (Banales et al., 2016). Furthermore, CCA cells may have different characteristic that can affect pathogenesis and outcome; for example, Li et al. (2016) identified down-regulation of FASN in intrahepatic cholangiocarcinoma while up-regulation of FASN was reported to enhance tumor aggressiveness in various cancer types including CCA, which is consistent with our study (Hao et al., 2014; Li et al., 2014; Yasumoto et al., 2016; Zaytseva et al., 2015; Zhang et al., 2020).

Taken together, FASN may serve as a potential target for the development of a novel CCA therapeutic strategy. The association of genes related to lipid metabolism, cell proliferation and metastasis should be examined to increase our understanding of the functions and targets of FASN. In addition, the cellular functions of FASN *in vivo* and knockout of FASN *via* CRISPR-Cas9 technology should be the subject of further study. Furthermore, the novel drugs, which can inhibit those lipid metabolizing enzymes especially FASN should be further investigation.

## Conclusion

The high expression of FASN was significantly correlated with advanced stage CCA, resulting in the shorter survival time of CCA patients. Furthermore, FASN knockdown inhibited the growth, migration, invasion, cell cycle and induced apoptosis in CCA cells. The study of metabolomics provides new insights into the mechanism associated with FASN knockdown in CCA cells and identified purine metabolism as the most relevant pathway. Targeting FASN may serve as a novel CCA therapeutic strategy.

## Data Availability

The original contributions presented in the study are included in the article/supplementary material, further inquiries can be directed to the corresponding author.
